# Chimeric Rabies Virus-Like Particles Containing Membrane-Anchored GM-CSF Enhances the Immune Response against Rabies Virus

**DOI:** 10.3390/v7031134

**Published:** 2015-03-11

**Authors:** Hongtao Kang, Yinglin Qi, Hualei Wang, Xuexing Zheng, Yuwei Gao, Nan Li, Songtao Yang, Xianzhu Xia

**Affiliations:** 1 College of Veterinary Medicine, South China Agricultural University, 483 Wushan Road, Guangzhou 510642, China; E-Mail: kang1989462@sina.com; 2 Institute of Military Veterinary Medicine, Academy of Military Medical Science, 666 Liuying West Road, Changchun 130122, China; E-Mails: qiyinglin1985@163.com (Y.Q.); wangh25@hotmail.com (H.W.); zhengxx2513@gmail.com (X.Z.); gaoyuwei@gmail.com (Y.G.); linan226@126.com (N.L.); 3 College of Veterinary Medicine, Jilin University, 5333 Xian Road, Changchun 130062, China

**Keywords:** rabies virus, virus-like particles, GM-CSF, rabies vaccine

## Abstract

Rabies remains an important public health threat in most developing countries. To develop a more effective and safe vaccine against rabies, we have constructed a chimeric rabies virus-like particle (VLP), which containing glycoprotein (G) and matrix protein (M) of rabies virus (RABV) Evelyn-Rokitnicki-Abelseth (ERA) strain, and membrane-anchored granulocyte-macrophage colony-stimulating factor (GM-CSF), and it was named of EVLP-G. The immunogenicity and protective efficacy of EVLP-G against RABV were evaluated by intramuscular administration in a mouse model. The EVLP-G was successfully produced in insect cells by coinfection with three recombinant baculoviruses expressing G, M, and GM-CSF, respectively. The membrane-anchored GM-CSF possesses a strong adjuvant activity. More B cells and dendritic cells (DCs) were recruited and/or activated in inguinal lymph nodes in mice immunized with EVLP-G. EVLP-G was found to induce a significantly increased RABV-specific virus-neutralizing antibody and elicit a larger and broader antibody subclass responses compared with the standard rabies VLP (sRVLP, consisting of G and M). The EVLP-G also elicited significantly more IFN-γ- or IL-4-secreting CD4^+^ and CD8^+^ T cells than the sRVLP. Moreover, the immune responses induced by EVLP-G protect all vaccinated mice from lethal challenge with RABV. These results suggest that EVLP-G has the potential to be developed as a novel vaccine candidate for the prevention and control of animal rabies.

## 1. Introduction

Rabies is one of the oldest zoonotic diseases that occurs worldwide and afflicts nearly all mammalian hosts. Once the infected, the host manifests clinical symptoms of rabies, the result is almost invariably lethal [1]. According to an estimate by the World Health Organization, rabies causes more than 55,000 human deaths and more than 15 million people undergo postexposure prophylaxis globally each year [2]. Most human cases occur in the developing countries of Africa and Asia, where bites from rabid dogs are the major cause [3]. Vaccination is still the most effective way to prevent and control rabies in animals and humans. There has been a dramatic reduction in human rabies cases in most developed countries due to mass the vaccination of domestic animals [4]. Despite the long history of rabies vaccine being used preventatively, more than two-thirds of the world’s population lives in regions where threat from rabies is high [5]. Therefore, the development of a more affordable, safe, and potent rabies vaccine is advisable and necessary.

Virus-like particles (VLPs) formed by one or several viral structural proteins are stable, non-replicative, non-infective, and highly immunogenic and have, therefore, become a novel safe option as a vaccine candidate [6]. VLPs can stimulate innate immunity by interacting with pathogen-associated molecular patterns and pattern recognition receptors, which further elicits adaptive immune and inflammatory responses against infection [7,8]. The VLPs of several viruses have been developed as effective vaccine candidates, such as human immunodeficiency virus (HIV), avian influenza virus (AIV), and human papilloma virus (HPV) [9,10,11].

The stimulation of antigen-presenting cells (APCs), especially dendritic cells (DCs), which are the most efficient of APCs, is considered as the key linkage between innate and adaptive immune responses against viral infection [12,13]. The recruitment and/or activation of DCs are important in inducing protective immunity [14]. Once the DCs are activated, they migrate to the lymphoid organs and then interact with B and T cells to initiate an adaptive immune response [15]. Granulocyte-macrophage colony-stimulating factor (GM-CSF) is a pleiotropic cytokine responsible for the proliferation, differentiation, and activation of macrophages, neutrophils, and various APCs [16,17,18]. It has been reported that GM-CSF has a variety of effects on immune responses, including increasing the antibody response and enhancing T cell proliferation [19]. The adjuvant activity of GM-CSF is partly mediated by chemo-attraction and activation of APCs, which triggers antigen internalization, processing and presentation to lymphocytes [20]. Furthermore, by increasing the numbers and maturation of DCs, GM-CSF enhances the immune responses to vaccines [21]. GM-CSF has been extensively used as an adjuvant in multiple vaccine platforms, including co-administration with DNA vaccines, basic live viral vector vaccines, and cancer immunogene therapies [22,23,24].

In our earlier studies, we observed that glycoprotein (G) and matrix (M) protein of rabies virus (RABV) can generate standard rabies VLP (sRVLP) by self-assembly in insect cells [25]. In this study we developed a chimeric rabies VLP (cRVLP) called EVLP-G that containing G and M of RABV Evelyn-Rokitnicki-Abelseth (ERA) strain and membrane-anchored murine GM-CSF, and characterized its composition, bioactivity and assembly properties. We evaluated the immune response and the protective efficacy induced by intramuscular (i.m.) immunization of EVLP-G in mice. Our results demonstrate that immunization with cRVLP containing GM-CSF is more efficient than sRVLP in inducing specific anti-RABV immune responses in a mouse model. Moreover, the protective immunity elicited by cRVLP was able to confer 100% protection against a lethal challenge with RABV street strain.

## 2. Materials and Methods

### 2.1. Cell Lines and Viruses

Sf9 cells (obtained from Prof. Deng from Harbin Veterinary Research Institute, Harbin, China) were maintained in suspension with SF900II serum-free medium (Life technologies, San Diego, CA, USA) at 27 °C in cell culture flasks at a speed of 120 rpm. HuNPB_3_, a RABV street strain, was isolated from a pig that died of rabies in the Hunan Province of China in 2006 and stored in our laboratory [26]. The RABV ERA strain (Accession NO. EF206707) was obtained from the China Veterinary Culture Collection.

### 2.2. DNA Construction and Recombinant Baculovirus (rBVs) Generation

We constructed a recombinant plasmid encoding a membrane-anchored GM-CSF gene consisting of a signal peptide (SP) from honeybee mellitin, full-length murine GM-CSF, and the transmembrane (TM) and cytoplasmic tail (CT) regions of G. All primers used for this study are listed in Table 1. The fragment containing mellitin SP and GM-CSF was amplified from the plasmid pMD-MSP-GMCSF (a kind gift from Dr. Zhiguang Ren) using primers MSP-GMF and MSP-GMR and then inserted into pFastBac Dual (pFBD, Life technologies) under the polyhedrin promoter, resulting in pFBD-SP-GM. The TM-CT region of G was amplified from cDNA of ERA using primers EG-TMCTF and EG-TMCTR and then inserted into pFBD-SP-GM to yield pFBD-GMCSF. Next, using pFBD-GMCSF as a template and SPGMF and SPGMR as primers, the PCR product was cloned into pFBD-GMCSF under the p10 promoter to obtain pFBD-2GMCSF, which contains two GM-CSF genes. The purified plasmid was transformed into DH10™Bac *E. coli* (Life technologies) for transposition into a bacmid. Then, a Cellfection^®^ II Reagent (Life technologies) was used according to the manufacturer’s instructions to generate the rBV rpFBD-2GMCSF. The rBVs rpFBD-2COG and rpFBD-2COM expressing G and M protein, respectively, were generated as reported previously [25]. Briefly, we constructed two recombinant plasmids pFBD-2COG and pFBD-2COM, which contained G and M genes from RABV ERA strain, respectively. The plasmids were transformed into DH10™Bac *E. coli* to obtain positive recombinant bacmids. Then, the bacmids were transfected into Sf9 cells to produce two rBVs rpFBD-2COG and rpFBD-2COM.

**Table 1:** Sequences of primers used in present study.

Primer	Sequence (5'-3')	Restriction Enzyme Site
MSP-GMF	TTTGGATCCATGAAGTTCCTGGTGAACGTGGCTC	BamHI
MSP-GMR	GGGGAATTCCTTTTGCACAGGCTTCTTGCACTCG	EcoRI
EG-TMCTF	CCCCGAATTCTATGTATTACTGAGTGCAGG	EcoRI
EG-TMCTR	TTTTAAGCTTTCACAGTCTGGTCTCACCCC	HindIII
SPGMF	GGGCTCGAGATGAAGTTCCTGGTGAACGTGGCTC	XhoI
SPGMR	TTTGCTAGCTTACAGGCGGGTCTCGCCACCGGAC	NheI

The sequences of restriction enzyme sites are underlined.

### 2.3. Immunofluorescence Assay (IFA)

Sf9 cells were infected with rpFBD-2COG, rpFBD-2COM or rpFBD-2GMCSF. At 48 h postinfection, cells were fixed with ice-cold 70% alcohol for 30 min at room temperature (RT) and blocked with 2% bovine serum albumin (BSA) for 60 min at RT. The cells were incubated with mouse anti-rabies G antibody (Millipore, Temecula, CA, USA), rabbit serum against RABV M or mouse anti-GM-CSF antibody (Abcam, Cambridge, MA, USA) for 90 min at 37 °C. Finally, the cells were stained with Alexa Fluor 488-conjugated goat anti-mouse or goat anti-rabbit IgG (Millipore, Boston, MA, USA) for 50 min at 37 °C and analyzed under a fluorescence microscope.

### 2.4. Production and Characterization of EVLP-G

To produce EVLP-G, which is essentially cRVLPs containing GM-CSF, Sf9 cells were coinfected with rBV expressing G, M and GM-CSF at multiplicities of infection of 3, 2, and 3, respectively, and incubated at 27 °C for 5 days. Culture supernatants were harvested and centrifuged at 2000 × *g* for 30 min to remove cells and then pelleted by ultracentrifugation at 30,000 × *g* for 60 min at 4 °C. The pellets were resuspended in PBS and purified through a 20%–40%–60% discontinuous sucrose gradient at 25,000 × *g* for 90 min at 4 °C. The EVLP-G band obtained between 40% and 60% density range was collected, washed, and resuspended overnight in PBS.

For Western blot analysis, EVLP-G and control sample (cell culture supernatant) were separated by 12% sodium dodecyl sulfate-polyacrylamide gel electrophoresis under denaturing conditions, transferred onto a nitrocellulose membrane (Whatman, Kent, UK) and then probed with rabbit serum against M, mouse anti-rabies G or mouse anti-GM-CSF antibodies at a dilution of 1:200 overnight at 4 °C. The sample was then incubated with horseradish peroxidase (HRP)-conjugated goat anti-mouse or anti-rabbit secondary antibody at a dilution of 1:4000 (Millipore, Boston, MA, USA) for 60 min at 37 °C.

For electron microscopy, EVLP-G was applied onto a carbon-coated formvar grid, which was immediately stained with 1% phosphotungstic acid and then observed by a transmission electron microscope. For immunoelectron microscopy, after binding EVLP-G to formvar-coated grids, which were sequentially incubated with mouse anti-rabies G or mouse anti-GM-CSF antibodies for 90 min at RT and gold-labeled goat anti-mouse IgG antibody (Sigma-Aldrich, Saint Louis, MO, USA) for 60 min at RT. Finally, the grids were stained with 1% phosphotungstic acid and examined under an electron microscope.

### 2.5. Immunization and Virus Challenge

Female BALB/c mice aged 6-8 weeks were purchased from Changchun Institute of Biological Products Co., Ltd, China. Mice were randomly divided into 3 groups and individually immunized twice with 10 μg/mouse EVLP (sRVLP, consisting of G and M), EVLP-G, or PBS by the i.m. route at two week intervals. At 4 weeks post the final immunization, mice were challenged i.m. with 100 × 50% intramuscular mouse lethal dose (IMLD_50_) of HuNPB_3_ in the muscle of the forelimb. The mice were observed for 21 days, any mice that developed clinical signs of rabies during the observation period were humanely euthanized by cervical dislocation under isofluorane anesthesia.

### 2.6. Antibody Assay

Blood samples were obtained by retro-orbital plexus puncture at 2 and 4 weeks. Serum levels of specific virus neutralization antibody (VNA) were measured using fluorescent antibody virus neutralization (FAVN) [27]. The serum (dilution is 5000) specific IgG, IgG1, IgG2a, IgG2b, IgG3 and IgM responses were examined using enzyme-linked immunosorbent assay (ELISA) [28,29]. Briefly, 96-well plates were coated with inactivated ERA and blocked with 2% bovine serum albumin. The diluted serum samples were added to each well and incubated. Following this, the plates were incubated with HRP-conjugated goat anti-mouse IgG, IgG1, IgG2a, IgG2b, IgG3 and IgM antibodies (Southern Biotechnology Associates, Birmingham, AL, USA). The substrate TMB (Sigma-Aldrich) was used to develop the color, and an ELISA reader was used to read the optical density at 450 nm.

### 2.7. IFN-γ and IL-4 Enzyme-Linked Immunospot Assays (ELISpot)

The spleens were collected from mice at 2 weeks after the second immunization and single splenocyte suspensions (2.5 × 10^6^ cells/mL) were prepared in complete RPMI 1640 medium (1640, Life technologies) with 10% fetal bovine serum (FBS, Life technologies). The cells were stimulated with inactivated ERA (The ERA strain mixed with β-propiolactone to a final concentration of 0.025% and then incubated at 4 °C overnight and at 37 °C for 2 h) at final concentration of 10 μg/mL and cultured for 24 h at 37 °C. The splenocytes producing IFN-γ or IL-4 were quantified by ELISpot assay (Mouse IFN-γ/IL-4 ELISPOT kit, Mabtech AB, Sweden) according to the manufacturer’s instructions. Spot-forming cells (SFCs) were enumerated using an automated ELISpot reader (AID GmbH, Strassberg, GER).

### 2.8. Flow Cytometry Assays for Intracellular Cytokine Staining (ICS)

At 2 weeks post-vaccination, isolated splenocytes at 1 × 10^7^ cells/mL in 1640 with 10% FBS were stimulated with inactivated ERA at a final concentration of 10 μg/mL and cultured in the presence of monensin (BD Biosciences, Franklin, TN, USA) at 37 °C for 6 h. After surface staining with anti-mouse CD4 and CD8 antibodies (BD Biosciences) for 30 min at 4 °C, the cells were permeabilized with Cytofix/Cytoperm (BD Biosciences) for 30 min at 4 °C and stained with anti-mouse IFN-γ and IL-4 antibodies (BD Biosciences) for 30 min at 4 °C. These stained cells were analyzed by a flow cytometer.

### 2.9. Flow Cytometry Assays for B Cells and DCs

The inguinal lymph nodes were collected at 3, 6, and 9 days after primary immunization. Single cell suspensions (1 × 10^6^ cells/mL) were prepared in PBS containing 2% FBS and stained with anti-mouse CD19, CD40, CD11_C_, CD80, CD86, MHC I, and MHC II antibodies (BD Biosciences) for 30 min at 4 °C (CD19 and CD40 for B cells and CD11_C_, CD80, CD86, MHC I, and MHC II for DCs) [23,30]. After staining, the labeled cells were washed twice with PBS containing 2% FBS and then analyzed in a flow cytometer.

### 2.10. Laboratory Facility and Ethics Statement

All animal studies were conducted with prior approval from the Animal Welfare and Ethics Committee of the Veterinary Institute at the Academy of Military Medical Sciences (permit number SCXK-2012-017). The environment and housing facilities satisfied the National Standards of Laboratory Animal-Requirements of Environment and Housing Facilities (GB 14925-2001) of China. Experiments involving the use of the RABV street strain were approved by the Military Veterinary Research Institute of the Academy of Military Medical Sciences and conducted in a biosafety level 3 laboratory.

## 3. Results

### 3.1. Construction and Generation of rBVs Expressing GM-CSF

**Figure 1:**
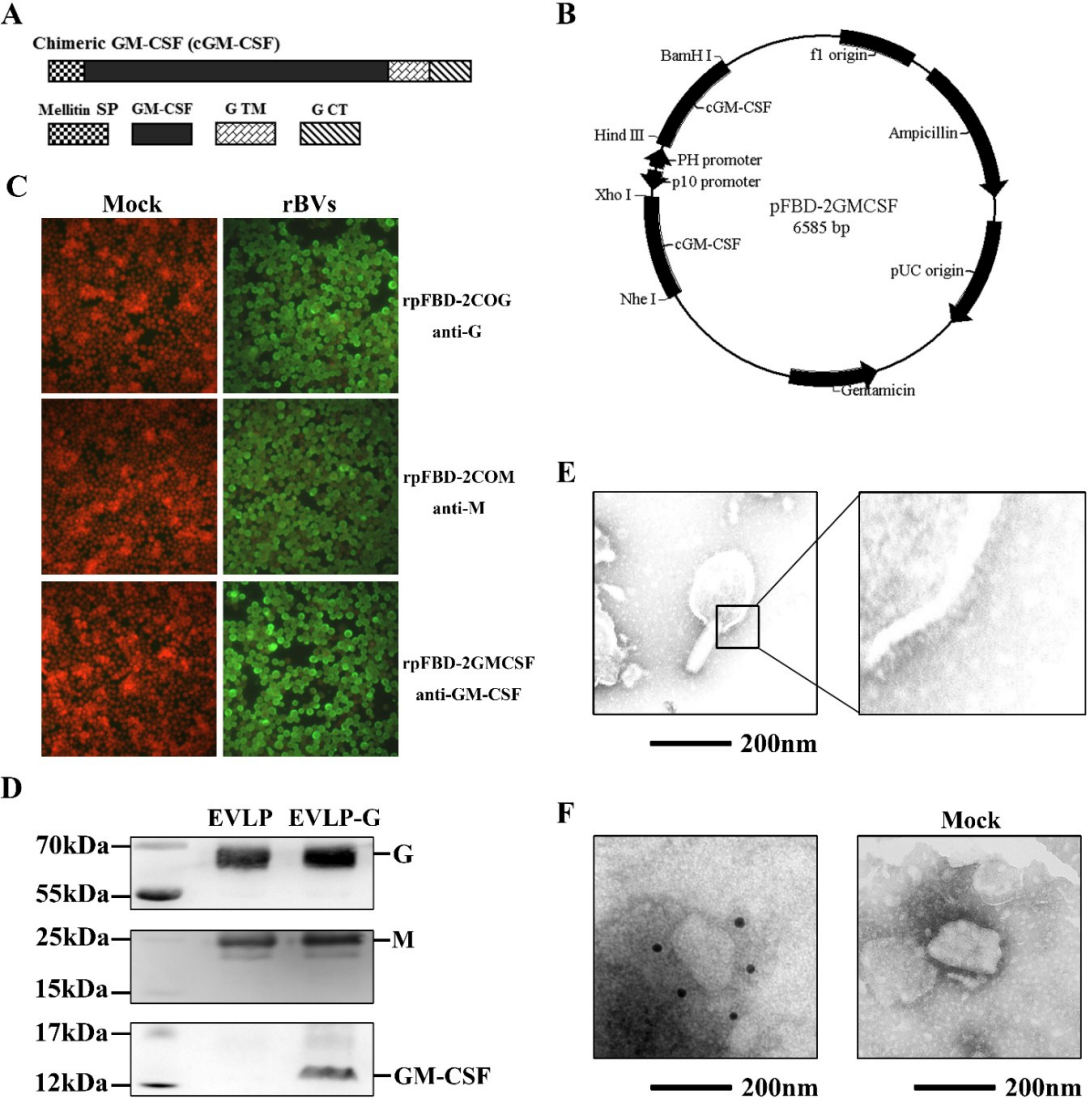
Construction and production of cRVLP containing membrane-anchored GM-CSF. (**A**) Schematic diagram of the construction about membrane-anchored GM-CSF containing mellitin SP and TM-CT of RABV G; (**B**) Schematic diagram of the recombinant plasmid pFBD-2GMCSF; (**C**) Detection of the expression of G, M and GM-CSF by rBVs. Sf9 cells were infected with rBVs rpFBD-2COG, rpFBD-2COM and rpFBD-2GMCSF, respectively. Additionally, the cells treated with PBS as mock. The infected cells were incubated at 27 °C for 48 h, and then examined the expression by using IFA with mouse anti-G antibody, rabbit serum against RABV M or mouse anti-GM-CSF antibody; (**D**) Western blot assays of EVLP-G. We analyzed purified EVLP-G for the incorporation of G, M and GM-CSF probed with mouse anti-rabies G antibody, rabbit serum against RABV M or mouse anti-GM-CSF antibody; (**E**) Electron microscopy of EVLP-G. The EVLP-G were stained with 1% sodium phosphotungstate and then observed by a transmission electron microscopy; (**F**) Immunoelectron microscopy of EVLP-G. The EVLP-G and EVLP (as mock) were incubated with mouse anti-GM-CSF antibody, and then with gold-labeled goat anti-mouse IgG antibody.

As depicted in Figure 1A, chimeric GM-CSF was constructed by fusing mellitin SP and TM-CT from the ERA G gene at the N terminus and the C terminus of full-length GM-CSF, respectively. A schematic of the recombination plasmid pFBD-2GMCSF containing two GM-CSF genes is shown in Figure 1B. The resultant rBVs, rpFBD-2COG, rpFBD-2COM, and rpFBD-2GMCSF, were rescued successfully in sf9 insect cells. Expression of G, M, and GM-CSF by the rBVs was detected by immunostaining. As expected, cells infected with rpFBD-2COG, rpFBD-2COM, or rpFBD-2GMCSF were stained by antibody against G, mouse serum against M, or antibody against GM-CSF, respectively (Figure 1C).

### 3.2. Production and Characterization of EVLP-G

EVLP-G particles were generated by coinfection with three rBVs expressing G, M, and GM-CSF, respectively. Western blotting analysis demonstrated that EVLP-G consists of G, M, and GM-CSF contents (Figure 1D). In addition, G-, M- and GM-CSF-specific bands were detected in the lane of control sample. To confirm the integrity and morphology of EVLP-G, the samples were examined by electron microscopy. As shown in Figure 1E, enveloped EVLP-G particles with a diameter of approximately 180–200 nm and with densely arrayed surface spikes were clearly visible. The morphology of EVLP-G was similar to sRVLP. To further confirm the incorporation of membrane-anchored GM-CSF, we performed an immunoelectron microscopy analysis. The results showed several gold particles located on the surface of EVLP-G when murine GM-CSF antibody was used as the primary antibody (Figure 1F). However, no gold particles were observed on the mock. These results indicate that membrane-anchored GM-CSF was incorporated into EVLP-G, and this incorporation did not change the size and morphology of rabies VLP.

### 3.3. Antibody Responses Induced by EVLP-G

**Figure 2:**
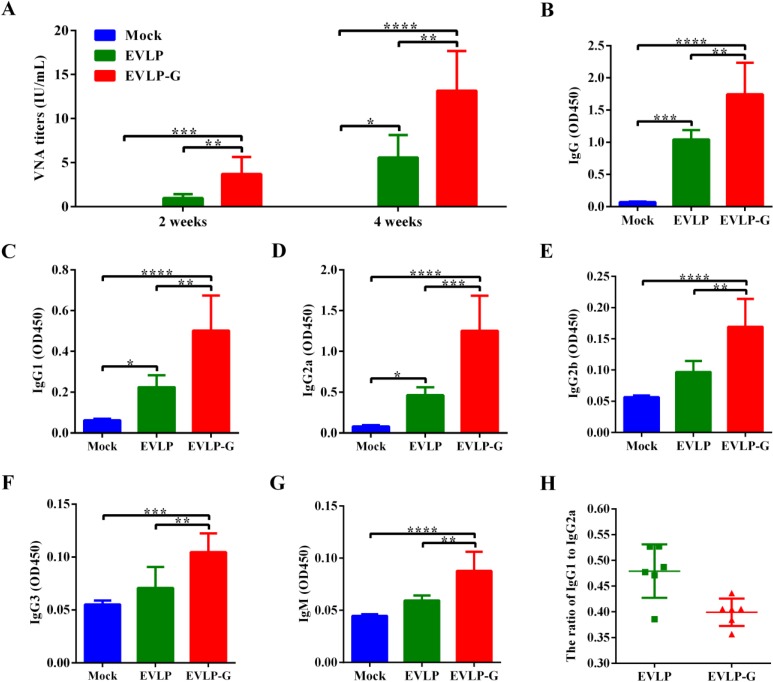
Specific anti-RABV antibody response induced by EVLP-G. Mice were immunizated twice with EVLP-G via i.m. route at 2-week intervals. (**A**) The production of specific anti-RABV VNA. Serum were collected at 2 and 4 weeks after vaccination, and the VNA titers were measured by FAVN; (**B**–**H**) The serum specific anti-RABV antibody subclass responses were detected as described in Material and Methods; The IgG (**B**), IgG1 (**C**), IgG2a (**D**), IgG2b (**E**), IgG3 (**F**) were determined at 2 weeks after the second immunization, while IgM (**G**) were evaluated at 7 days after the first immunization; And the ratio of IgG1/IgG2a (**H**) were measured. Representative data are the mean ± standard deviation (SD) of 8 mice from each group and were analyzed by one-way ANOVA (* *p* < 0.05, ** *p* < 0.01, *** *p* < 0.001, **** *p* < 0.0001).

To evaluate the immunogenicity of EVLP-G in a mouse model by using the vaccination schedule as described in Materials and Methods. Figure 2A shown the VNA titers in mice immunized with EVLP, EVLP-G or PBS. At two weeks after the first immunization, specific VNA were detected in serum from all mice treated with EVLP or EVLP-G, and the means of VNA titers were 0.96 IU/mL and 3.68 IU/mL, respectively. Mice were administered the second immunization at two weeks. This resulted in a significant increase in the VNA responses, and the titers against RABV rose to 5.57 IU/mL and 13.16 IU/mL, respectively. The VNA titer in EVLP-G groups was significantly higher than that in the EVLP group after immunization. These data suggest that immunization with EVLP-G containing membrane-anchored GM-CSF induced a significantly enhanced specific anti-RABV VNA response in mice compared with sRVLP immunization.

To further investigate whether the pattern of IgG subclasses induced by EVLP-G containing GM-CSF could be changed or/and broadened, the specific serum IgG and subclass responses were determined by ELISA. As shown in Figure 2B–G, the total IgG and IgG1, IgG2a, IgG2b, IgG3, and IgM subclass antibody response induced by EVLP-G was significantly higher than those of EVLP or PBS. Moreover, compared with PBS, EVLP did not induce an enhanced IgG2b, IgG3 and IgM response. As shown in Figure 2H, EVLP and EVLP-G induced both Th1 and Th2 immune responses. However, the ratio of IgG1/IgG2a (Figure 2H) indicating that they both elicited a Th1-biased type-mixed response. These results suggest that EVLP-G elicits a stronger and broader antibody subclass response in mice, and membrane-anchored GM-CSF does not change the dominant antibody response.

### 3.4. Antigen-Specific Cellular Immune Responses Induced by EVLP-G

**Figure 3:**
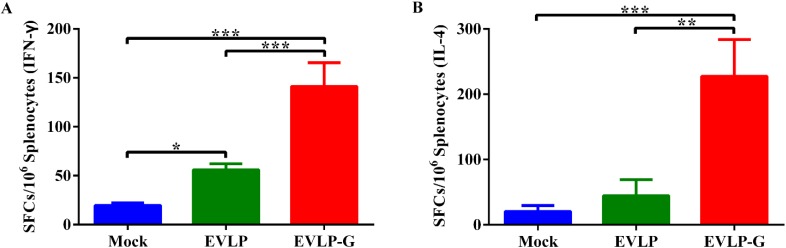
ELISpot analysis of IFN-γ and IL-4 secretion in mouse splenocytes. The splenocytes were collected from each group 2 weeks after the second immunization, and were treated and analyzed as described in Material and Methods. The secretion of IFN-γ (**A**) and IL-4 (**B**) were measured by ELISpot Kit. The data represent the means of SFCs per million splenocytes from 3 mice of each group with SD, and were analyzed by one-way ANOVA (* *p* < 0.05, ** *p* < 0.01, *** *p* < 0.001).

After confirming that EVLP-G successfully induced enhanced VNA response in mice, we next evaluated the antigen-specific IFN-γ and IL-4 activities in splenocytes by ELISpot assays. As shown in Figure 3A,B, the SFCs of IFN-γ and IL-4 from the splenocytes of mice immunized with EVLP-G were significantly more than those of EVLP- or PBS-immunized mice. Compared with the PBS group, EVLP hardly induced any IL-4 response in mice, illustrating that the antigen-specific IFN-γ and IL-4 responses induced by EVLP-G significantly exceeded those induced by sRVLP.

**Figure 4:**
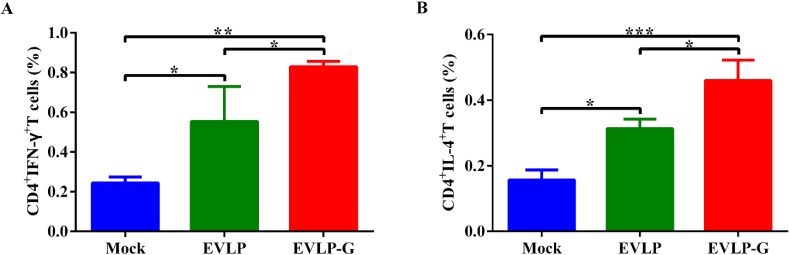

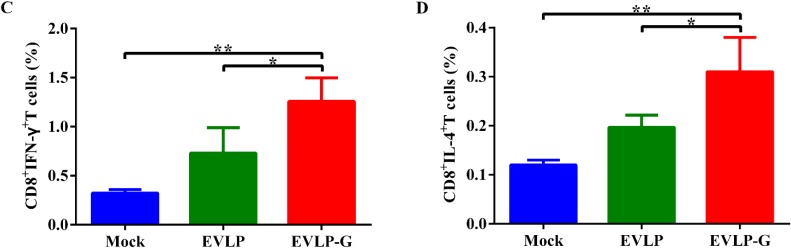
ICS assays for antigen-specific CD4^+^ and CD8^+^ T cells secreting IFN-γ or IL-4. The spleen from 3 mice of each group were isolated 2 weeks after the last vaccination. The splenocytes were prepared, stimulated, and cultured as described in the Material and Methods. Then the splenocytes were stained with mouse anti-CD4, -CD8, -IFN-γ, and -IL-4 monoclonal antibodies. The CD4^+^ T cells secreting IFN-γ (**A**) or IL-4 (**B**); and the CD8^+^ T cells secreting IFN-γ (**C**) or IL-4 (**D**) were shown. The data represent the means of subtraction values with SD and were analyzed by one-way ANOVA (* *p* < 0.05, ** *p* < 0.01, *** *p* < 0.001).

To further characterize the T cell’s response induced by rabies VLPs, we evaluated the ability of EVLP or EVLP-G to induce IFN-γ- or IL-4-secreting CD4^+^ and CD8^+^ T cells. As shown in Figure 4A,B, both EVLP and EVLP-G elicited an apparently enhanced IFN-γ- or IL-4-secreting CD4^+^ T cell response compared with PBS treatment, but the percentage of CD4^+^IFN-γ^+^ or CD4^+^IL-4^+^ cells was significantly higher in the EVLP-G group than that in the EVLP group. As shown in Figure 4C and 4D, the number of CD8^+^ T cells secreting IFN-γ or IL-4 induced by EVLP-G was significantly more than the EVLP or PBS induction. The difference between EVLP and PBS groups was not statistically significant. These data demonstrated that mice immunized with EVLP-G containing GM-CSF elicited a notably enhanced IFN-γ- or IL-4-secreting CD4^+^ and CD8^+^ T cell response.

### 3.5. EVLP-G-Induced Recruitment and/or Activation of B Cells and DCs in Lymph Nodes

**Figure 5:**
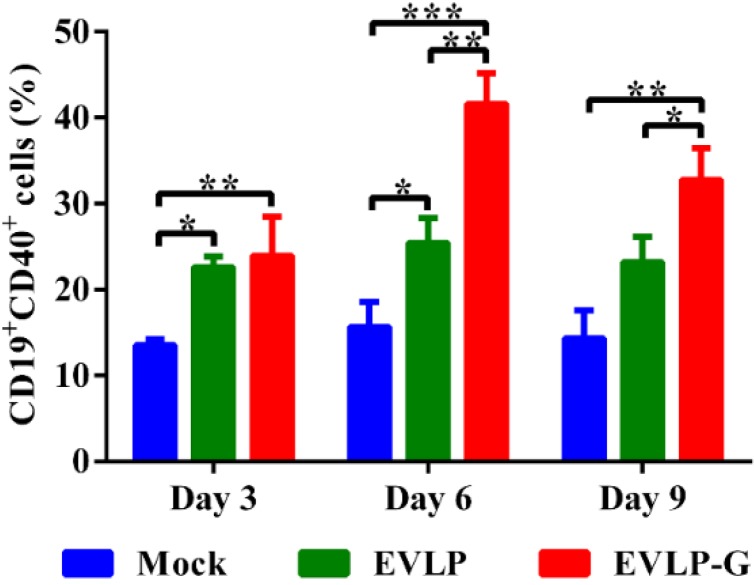
Flow cytometry assay of recruitment and/or activation of B cells in inguinal lymph nodes. The inguinal lymph nodes were collected from mice immunized with EVLP, EVLP-G and PBS (*n =* 3) on 3, 6, and 9 days after the first immunization. The cells in lymph nodes were stained with mouse anti-CD19, -CD40 monoclonal antibodies. The double positive cells of CD19^+^CD40^+^ were plotted. The data represent the means of double positive cells percentage with SD and were analyzed by one-way ANOVA (* *p* < 0.05, ** *p* < 0.01, *** *p* < 0.001).

**Figure 6:**
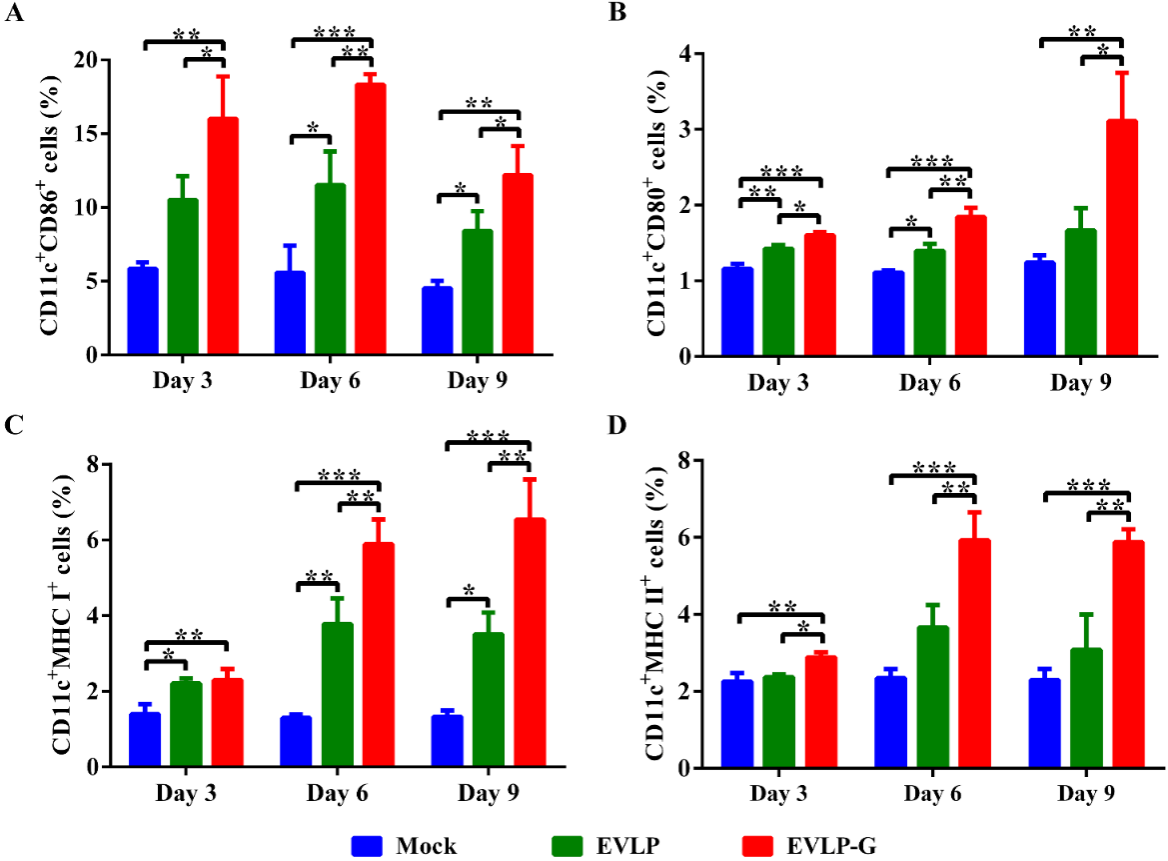
Flow cytometry assay of recruitment and/or activation of DCs in inguinal lymph nodes. The inguinal lymph nodes were collected from mice immunized with EVLP, EVLP-G and PBS (*n =* 3) on 3, 6, and 9 days after the first immunization. The cells in lymph nodes were strained with mouse anti-CD11C, -CD80, -CD86, -MHC I, and -MHC II monoclonal antibodies. The double positive cells that CD11c^+^CD86^+^ (**A**); CD11c^+^CD80^+^ (**B**); CD11c^+^MHC I^+^ (**C**); and CD11c^+^MHC II^+^ (**D**) were plotted. The data represent the means of double positive cells percentage with SD and were analyzed by one-way ANOVA (* *p* < 0.05, ** *p* < 0.01, *** *p* < 0.001).

To investigate whether membrane-anchored GM-CSF incorporated into cRVLP can function as an adjuvant and induce more B cells and DCs, lymph node cells were analyzed by flow cytometry. As shown in Figure 5, immunization with EVLP or EVLP-G induced more B cells (CD19^+^CD40^+^ cells) in lymph nodes than PBS alone. However, the percentage of CD19^+^CD40^+^ cells in mice vaccinated with EVLP-G was significantly higher than that induced by EVLP at six and nine days after immunization. As shown in Figure 6A,B, significantly more DCs (CD11c^+^CD86^+^ and CD11c^+^CD80^+^ cells) were detected at all time points in lymph nodes from mice vaccinated with EVLP-G compared with mice immunized with EVLP or mice treated with PBS alone. The expression of MHC I and MHC II on the surface of DCs is shown in Figure 6C,D, respectively. Compared with PBS, both EVLP and EVLP-G induced high levels of MHC I expression in vaccinated mice, but markedly higher levels of MHC I were detected in the EVLP-G group compared with the EVLP group at six and nine days after immunization. The difference in MHCII expression was not statistically significant between the EVLP and PBS groups, but DCs in mice immunized with EVLP-G expressed significantly higher levels of MHCII than did EVLP- or PBS-immunized mice. Taken together, these results indicate that EVLP-G containing membrane-anchored GM-CSF can induce enhanced recruitment and/or activation of B cells and DCs in inguinal lymph nodes, and also elicit higher levels of MHC I and MHC II expression on the surface of activated DCs compared with sRVLP.

### 3.6. Challenge Test

**Figure 7:**
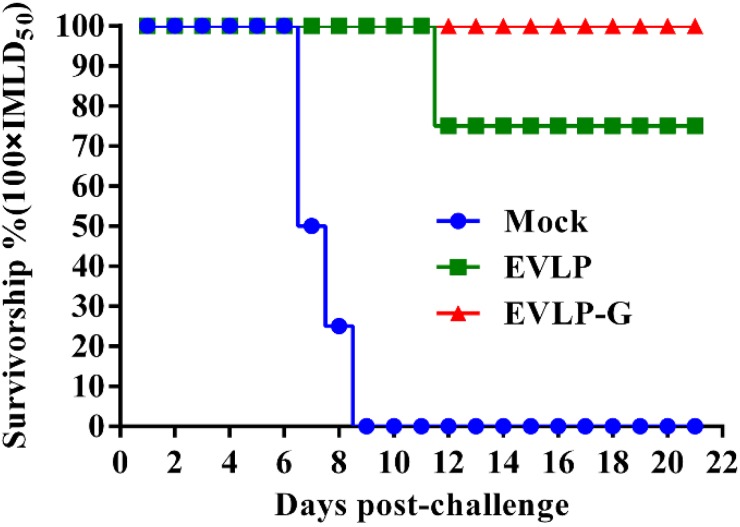
Challenge test in mice. All mice from each group (*n =* 8) were challenged with 100 × IMLD_50_ of the RABV street stain at 4 weeks after the last vaccination by i.m. route, and the observed for 21 days. The survival of mice in each group at different times after challenge were recorded.

To further evaluate whether the immune response induced by EVLP-G can protect against RABV, mice were challenged with 100 × IMLD_50_ RABV street strain (Figure 7). All mice mock immunized with PBS died of rabies within nine days of exposure to RABV challenge. In the EVLP-immunized group, two mice developed typical clinical symptoms of rabies and were humanely sacrificed at 12 days after infection, resulting in a 75% survival in that group. However, all mice from EVLP-G group were successfully protected against the high dose RABV challenge, and no clinical signs of rabies were observed in these mice during the 21-day observation period. RABV antigens were detected in the brains of mice that died from the challenge. The results of challenge experiment demonstrated that the immune responses induced by EVLP-G in mice can provide complete protection against a lethal challenge with high dose of RABV street strain.

## 4. Discussion

A wide variety of VLP-based vaccine candidates have been generated in different expression systems. Some of them have entered clinical development, and a few have been licensed and commercialized. The antigenic epitopes of viruses were conformed correctly and displayed on the surface of VLP in a highly repetitive manner. VLP as exogenous antigens are efficiently taken up by APCs, like the DCs, which process and present VLP antigen fragment on MHC II to lymphocytes. The expression of co-stimulatory molecules and the secretion of cytokine promote DCs activation and maturation, and stimulation of CD4^+^ Th cells, result in the production of humoral and cellular immune responses [6]. VLP also processed in the cytosol of DCs like a native virus and presented by MHC I to cytotoxic CD8^+^ T cells, which called “cross-presentation” mechanism and inducing potent cytotoxic immune response [31]. Although VLP elicit less robust immune responses than attenuated live vaccines, they are more immunogenic than subunit vaccines.

We had previously shown that rabies VLPs could be produced in insect cells and are able to induce an immune response against RABV challenge in mice [25]. Based on these observations, we explored the possibility of further improving the quality and quantity of rabies VLP-induced immune response. One such strategy was to modify the sRVLP with the immunostimulatory molecule GM-CSF. It has been demonstrated that the amount of recombinant foreign protein expression from the baculovirus/insect cells system can be increased by using signal peptides of insect origin [32]. Therefore, we chose the honeybee mellitin SP, which is known to improve the expression of cell surface glycoproteins in insect cells [33]. In addition, the sequence of TM-CT from RABV G was used as a membrane anchored domain to engage the modified GM-CSF to assemble into cRVLP. Using this strategy, we observed that membrane-anchored GM-CSF gene was well expressed in Sf9 insect cells and was successfully incorporated into cRVLP. Importantly, this incorporation did not alter the morphology of rabies VLPs. The rabies VLPs in our study do not take on typical rhabdovirus morphology. The reason may be that the absence of support from and connection to internal ribonucleoprotein causes the RVLPs to exhibit an atypical bullet-shaped structure.

GM-CSF is an important hematopoietic growth factor and immune modulator. The biological activities of GM-CSF have been used to enhance vaccine-induced immune responses [30,34,35]. In this study, the membrane-anchored GM-CSF incorporated into cRVLP was exploited for its apparent adjuvant activity. EVLP-G induced a significantly enhanced specific anti-RABV VNA response and a remarkably stronger, broader and more balanced antibody subclass response in mice compared with sRVLP. The IgG1 response was associated with a Th2 profile, while other subclasses were primarily associated with a Th1 immune response [36]. Similar to sRVLP, EVLP-G induced relatively high levels of IgG2a and resulted in a low IgG1/IgG2a ratio, which demonstrated that EVLP-G triggered a Th1-biased response pattern that is preferentially involved in cell-mediated immunity. These data suggested that rabies VLP membrane-anchored GM-CSF has a Th1-inducing adjuvant activity. High serum IgG3 titers have been demonstrated to mediate HIV neutralization in HIV-infected individuals [37], and is known to mediate important protective biological functions such as complement fixation, opsonization and induction of antibody-dependent cell cytotoxicity by natural killer cells [38]. Interestingly, significantly high IgG3 titers were detected in mice immunized with EVLP-G, indicating that the specific high levels of IgG3 might lead to a faster and more effective clearance of RABV via the mechanisms listed above. IgM is the first antibody produced after infection or immunization and develops a high valency because of its pentameric structure [39]. EVLP-G also induced an enhanced IgM response, which helps to prevent the spread of RABV from the sites of entry to the central nervous system resulting in protection within days of vaccination while higher affinity IgG antibodies are being formed in germinal centers [40]. The significantly enhanced specific VNA and antibody subclass responses induced by EVLP-G containing membrane-anchored GM-CSF in mice provided complete protection against the lethal challenge from high dose RABV street strain.

The recognition of antigenic peptides displayed by MHC molecules on APCs initiates the adaptive immune response. As critical specialized APCs, DCs are positioned throughout the body to detect environmental and pathogenic threats from a large variety of microorganisms [41]. DCs play an important role in stimulating the proliferation and differentiation of naïve and memory T cells [42]. GM-CSF is a cytokine important for the recruitment, activation and maturation of APCs, resulting in increased antigen presentation and further enhancement of the immune responses [35]. In this study, we found that GM-CSF incorporated into cRVLP in a membrane-anchored form demonstrated an adjuvant activity, which induced remarkably more DCs recruitment and/or activation (including DC11c^+^CD86^+^ cells and CD11c^+^CD80^+^ cells) in the inguinal lymph nodes of EVLP-G-immunized mice. In combination with CD28, the co-stimulatory markers CD86 and CD80 expressed on mature DCs deliver a signal to the T cells, which is often referred to as the second signal and is essential for the induction of effector Th cells [43]. EVLP-G also induced significantly higher levels of MHC I and MHC II expression on activated DCs compared with sRVLP. The major cellular pathways of antigen processing and presentation involve MHCI and MHCII. This suggests that more efficient uptake of EVLP-G by DCs as a result of increased MHCII expression and subsequent activation of co-stimulatory molecules and cytokines can lead to enhanced humoral and cellular immune responses. Similarly, increased expression of MHC I molecules, which is involved in a process called “cross-presentation”, can generate robust protective cytotoxic T lymphocyte (CTL) response [31].

Based in the type of co-receptor expressed on their surface, T lymphocytes have been divided into CD4^+^ T and CD8^+^ T cells, which mediate antigen-specific cellular immune responses. Flow cytometry analysis for ICS showed that EVLP-G containing GM-CSF functions as an adjuvant and activates significantly more IFN-γ- or IL-4-secreting CD4^+^ T cells. IFN-γ secreted by Th1 cells activates macrophages, thereby increasing the killing of phagocytosed microbes. On the other hand, IL-4 produced by Th2 cells drives the maturation of B cells into plasma cells, resulting in antibody production, isotype-switching and affinity maturation [44]. The results presented here show that significantly more activated B cells were recruited in inguinal lymph nodes in EVLP-G-immunized mice, which in turn induced a higher and broader serum antibody subclass response. Moreover, these mice also presented a notable increase in IFN-γ- or IL-4-secreting CD8^+^ T cells. The CD8^+^ T cells further differentiate into CTLs to kill the infected cells [31]. These results strongly indicated that cRVLP membrane-anchored GM-CSF acts as an adjuvant to stimulate both Th1 and Th2 type responses, as well as CD8^+^ T cells responses. However, further knowledge of detailed mechanisms including the expression and regulation of relevant cytokine needs further research.

The most important characteristic of a good vaccine candidate, especially in a non-infectious and non-replicative form, is its immunogenic potential. In this study, the immunogenicity of EVLP-G containing membrane-anchored GM-CSF was found to be much higher compared with sRVLP. The significantly higher RABV-specific VNA titers was detected in EVLP-G-immunized mice. Moreover, EVLP-G also induced a stronger CD4^+^ T and CD8^+^ T cell response. Both arms of the immune response mentioned above resulted in complete protection when the mice were challenged with a high dose of lethal RABV street strain. Taken together, the increased immunogenicity, stronger immune response, and improved protection against RABV infection underscored the potential for EVLP-G to be developed as a novel vaccine candidate for the prevention and control of animal rabies.
